# Preventive medicine for anorexia of female adolescent

**DOI:** 10.1192/j.eurpsy.2021.1853

**Published:** 2021-08-13

**Authors:** L. Bracco

**Affiliations:** Scientific Director Of Lorenzo Bracco Foundation, Lorenzo Bracco Foundation, Torino, Italy

**Keywords:** Anorexia, Blood Types (0; A; B; AB), preventive medicine, Female Adolescent

## Abstract

**Introduction:**

Anorexia of females adolescents has a high mortality rate and heavy health, psychological, family consequences even in case of survival.

**Objectives:**

To reduce the mortality rate and the consequences of anorexia by providing a theory that allows us to have early or even predictive diagnosis

**Methods:**

25 years ago I found blood type (O, A, B, AB) difference between an anorexic patient and her mother. Pregnancy had been with placental detachment and birth was traumatic, presumed causes of a mother/daughter blood contact. From that day on, I checked, in the case of Anorexia of the Female Adolescent, the blood types of the anorexic girl and her mother.

**Results:**

In my collection of data (more than 100 cases in 25 years): only the girls who have a different blood type (O, A, B, AB) from the mother are anorexic and from the patient’s history, we could think of a mother/daughter blood contact during the pregnancy. There are no exceptions in my data. My new theory is that Anorexia of the Female Adolescent, in addition to the girl’s psychological causes, needs a “conditio sine qua non” (a necessary but not sufficient condition): Different mother/daughter blood types (O,A,B,AB) and traumatic contact between the two blood types during pregnancy and/or birth”.

**Conclusions:**

My theory facilitates early diagnosis (Preventive Medicine) by limiting observation, for Anorexia risk, to only daughters with a different blood type than that of the mother. Recognizing this “conditio sine qua non” for Anorexia of the Female Adolescent allows us an early diagnosis and a predictive hypothesis.

**Disclosure:**

No significant relationships.

